# Access, understanding, promotion and maintenance of good health: Evaluation of knowledge transfer of people with intellectual disabilities to bridge the health information and disease prevention in public health

**DOI:** 10.3389/fpubh.2022.915970

**Published:** 2022-09-27

**Authors:** Lisa T. Dam, Petra Heidler, Isabel King

**Affiliations:** ^1^International Cooperative Cross-Border Interdisciplinary Doctoral Programme in Educational and Communication Sciences, University of Applied Sciences Burgenland, Eisenstadt, Austria; ^2^Department for Economy and Health, University for Continuing Education Krems, Krems an der Donau, Austria; ^3^Department of Health Sciences, St. Pölten University of Applied Sciences, Saint Pölten, Austria; ^4^Department of Exercise Physiology, School of Health and Behavioral Sciences, University of the Sunshine Coast, Sunshine Coast, QLD, Australia

**Keywords:** knowledge transfer, health information, intellectual disabilities, health inequalities, health literature, accessibility, easy-to-read language, communication barriers

## Abstract

The importance of patient empowerment among people with intellectual disabilities (ID) is steadily growing, yet multiple health and health literacy challenges still make this population more vulnerable to health disparities. Inadequate access to essential health and other basic services and the lack of involvement in health and educational research are some of the most crucial factors contributing to this inequality. A greater effort must be made to include people with ID in health literacy and communication research, preliminary focusing on language, including pictorial language. This community case study aimed to document the experiences of answering a pilot questionnaire to evaluate the problems of people with mild to moderate ID, 6A00.0 and 6A00.1 according to ICD-11. Our results show that most patients acquire health information from their physician or a medical professional. In preparing appropriate questionnaires, special education teachers can give valuable insight. However, participants were easily distracted and needed support in focusing on the questionnaire. Easy-to-read language, a simple format, big fonts and the presence of confidential caretakers are needed. This indicates, that semi-structured interviews with a trained interviewer might be most suitable for measuring the knowledge transfer of people with ID. The results of this case study highlight the need to develop an appropriate questionnaire and emphasize the need for a continued dialogue between people with ID and healthcare providers.

## Introduction

Approximately one percent of the Austrian population are intellectually disabled (1). Persons with ID are more likely to have physical disabilities, hearing impairments, vision impairments, mental health problems and communication disorders. These co-existing impairments, combined with limited intellectual functioning and adaptive behaviors, make this population particularly vulnerable to health disparities (2). Government reports outline two major problems in the accessibility of health information for people with disabilities. Firstly, the complexity of the written information is a significant barrier to comprehension (1, 3), and secondly, the communication barriers between people with intellectual disabilities and health professionals, which may result in a poorer state of health of the party concerned (4). Therefore, accessibility of health information and an adequate knowledge transfer has to be created to improve the health literacy of people with ID and reduce inequalities in that context.

Moreover, cognizant of the accessibility problem and the increasing importance of patient empowerment, people with ID are still underrepresented in health education and health literacy research (5). Digital technologies, such as mobile devices, mobile applications, and online resources, are useful tools to enhance health and digital literacy and monitor access, utilization, and impact of health-related knowledge transfer (6, 7). For example, aids like Web Content Accessibility Guidelines and “Easy to Read” (“Leicht Lesen^1^”) can help to improve the accessibility of information provided (3). Another tool to simplify the comprehensive process is “Photovoice,” a visual research methodology that puts cameras into the participants' hands to help them record, reflect upon and communicate issues of concern (5, 8). “Photovoice” is commonly used in the research fields of education, community development and public health, as it is a valid tool to make the process of knowledge transfer more inclusive (5, 8, 9). This case study aimed to evaluate the health education and literacy of people with intellectual disabilities in Austria, focusing on language, including pictorial language, by evaluating a pilot questionnaire. The wider objective of this evaluation is to reflect on how a questionnaire needs to be designed to measure knowledge transfer in health-related topics using media technologies, focusing on people with intellectual disabilities. With the integration of the information and insights gained from this case study, an adequate questionnaire will be developed to make the process of health information transfer and comprehension more inclusive.

## Context, background and rationale

The World Health Organization described intellectual disability (ID) as a significantly reduced ability to understand new or complex information and learn and apply new skills (impaired intelligence), resulting in a reduced ability to cope independently (impaired social functioning) (10). ID conditions originate before the age of 18, with a lasting effect on development over the lifespan (2). Individuals with mild to moderate ID are classified as 6A00.0 and 6A00.1 according to ICD-11 or F70 and F71 regarding the previous ICD-10 (11, 12). These conditions are characterized by “*below average intellectual functioning and adaptive behavior*” and difficulties in elaborating language and learning (12). Furthermore, difficulties in accessing education and healthcare facilities and inequity within these settings may result in limited employability, lower income and poorer health (13). In addition, the lack of inclusion in daily life and research results in the less ideal current situation of people with ID (4, 5).

Regarding the poverty report 2019 (13), no coherent framework for actions designed to support and implement the inclusion of people with intellectual disabilities has yet been developed. At least in parts, this may be explained by the lack of understanding and information about the scope and dimension of policy issues. European and national census data make it difficult to draw a clear picture of the number of persons with intellectual disabilities and identify households, including those with an intellectual disability (13). References to a disability should be included in the indicators used to evaluate social inclusion policies. With the existing General Data Protection Regulation (GDPR), the situation in Austria deteriorated as entry barriers have been raised and hindered access. Therefore, to improve the overall health of this population group, further involvement and consideration in research, especially health education research, is paramount to enhance the situation of people with intellectual disabilities.

Knowledge transfer, defined as “the process of communicating knowledge that has been developed in one part of an organization to other parts of the organization or customers” (14), is commonly used for transfers between educational and/or industrial organizations, experts and non-experts as well as between different academic disciplines. Knowledge transfer also includes educational programs for a lower social class, such as people with intellectual disabilities (ID). The challenge for measuring knowledge is that it is not bound to one part of the organization as it resides within the individuals, the organization's culture, workplace, standard operating processes, and structure. Most commonly used techniques for measuring knowledge, such as questionnaires or verbal protocols, asses changes in knowledge embedded in individuals, although it may be necessary to measure the changes in knowledge and performance in all parts of the organization (15).

## Materials and methods

A qualitative approach with a pilot questionnaire was chosen. Qualitative research spotlights and develops a detailed understanding of participants' individual experiences. It is unconcerned with sample representativeness; therefore, it does not apply a priori sample size estimation (16, 17). In addition (16, 17), previously discussed pre-determined sample size and saturation in qualitative research and argued that this decision should be made during the development of the progressively “comprehensive picture” of the research, as it depends on the researched topics. For this pilot project, convenience sampling was applied. Convenience sampling, Haphazard sampling or Accidental sampling is a non-probability or non-random sampling type where the target population is included as it meets practical criteria for example easy accessibility, geographical proximity, availability at a given time, or the willingness to participate the study (18). Researching subjects of the population that are easily accessible to the researcher, it is affordable and subjects are readily available (19, 20).

The Federal Government of Lower Austria works with non-profit organizations or companies offering sheltered workshops or sheltered employment according to the Employment of People with Disabilities Act (Disability Employment Act). The Federal Disability Act, the Federal Disability Equality Act, and the Disability Employment Act form the legal basis for disability law. Caritas Lower Austria West is a partner organization of the Federal Government of Lower Austria, leading 16 facilities in the region. The facility managers have approved all interviews. In cooperation with caretakers' people with ID working at the workshops have been asked if they are willing to participate the study. Non-probability sampling methods (e.g., convenience sampling, quota sampling, & snowball sampling) address individuals as they can furnish elaborate inputs to address the questions under investigation estimation (16, 17).

Ten people with intellectual disabilities, ranging from mild to moderate severity, participated in this community case study. All content and procedures were comprehensively explained to participants by their caretakers before the commencement of the study. Consent to participate was given, ensuring all participants that they could withdraw from the study at any time. The intellectual disability of the target group restricts itself 6A00.0 and 6A00.1, a mild and moderate disorder of intellectual development according to ICD-11 or F70 to F71 according to the previous ICD-10 (11, 12, 21). To be included, participants needed to be able to read and understand the content, have a basic interest in health-related topics and be able to use the internet. Upon commencing with the questionnaire, investigators explained the questionnaire's aim and familiarized each participant with the website. Participants were then asked to fill out a pilot questionnaire using the LUCHS website to answer the questions. A social worker was present to support the individuals during the entire process. The study was carried out in a local social aid facility.

The adapted pilot questionnaire used in this case study was based on previously developed online surveys to assess the knowledge transfer and health literacy in this population (22–24). Since it was a pilot test, and the first time introduced in this specific population, reliability or validity tests have not been taken into account. An earlier pilot test by Reeves et al. (25) evaluated the existing Picker test in order to determine if important items were missing. We orientated ourselves toward existing questionnaires and adapted the questionnaire according to the specification of the study. Van Teijlingen and Hundley (26) highlight the importance of pilot tests and stress the need for more discussion about their outcomes among researchers and highlighted the need for increased dialogues about the conduction of pilot studies themselves before further testing of revised questionnaires or tools, including reliability and validity tests, takes place. The pilot questionnaire was developed to assess the usability and measure the transfer of knowledge acquired *via* the health information website “LUCHS Gesundheits-Informationen” to identify the needs of people with disabilities and design an appropriate questionnaire with the gained knowledge. The aim of LUCHS (German for lynx) was to create a website containing health information tailored to the interests of the participants. The website's topics entailed nutrition, medical specialization, first aid and patient information and were related to the topics of the pilot questionnaire since the website served as a base for knowledge transfer. Non-participating people with intellectual disabilities proofread the website content and tested its usability. The content was then adapted according to their feedback. The participants had access to the website before the team introduced the questionnaire. As a next step and to accommodate visional impairment, the questionnaire was printed in A3 landscape format using a simple layout and a sans-serif font. In accordance with recent research, the structured questionnaire instrument was considered. As proposed by Curtin et al. (27), the structured interview provides an important opportunity to learn directly.

Moreover, a structured questionnaire provides initial insight into people with intellectual disabilities' participation in research (28) and allows the collection of information about perceived needs (29). Especially for individuals with restricted communication skills, structured support is assistant (30). The usage of visual media provides alternative ways to present meanings and concepts in an interview situation (31). The questionnaire comprised 14 questions divided into three parts and across four domains of knowledge transfer: demographic data, computer and Internet usage information, health information acquisition, and knowledge transfer. All questions, except one demographic question, were closed questions with dichotomous answers. The eight questions addressing knowledge transfer in the four categories are outlined in Table 1.

**Table 1:** Eight questions that participants with ID were asked across four domains of health-related knowledge transfer.

Nutrition	1a	Water is the healthiest beverage. Are sugar-free teas also healthy beverages?
	1b	Does cheese supply proteins for our body?
Medical specialization	2a	Does one need to see an orthopedist if one suffers from earache?
	2b	Does a dermatologist perform allergy tests?
Patient information	3b	I know that I am registered in ELGA (Austrian Electronic Health Record). Is it possible to deregister if I do not want to be registered in ELGA?
	3a	Does an occupational therapist help patients with reducing weight?
First aid	4a	Burns are labeled in four degrees. Is it necessary to visit a physician if one suffers from second-degree burns?
	4b	Take a look at the picture below (*image of a man providing CPR displayed*). Do you know what the man is doing?

## Results

**Figure 1:**
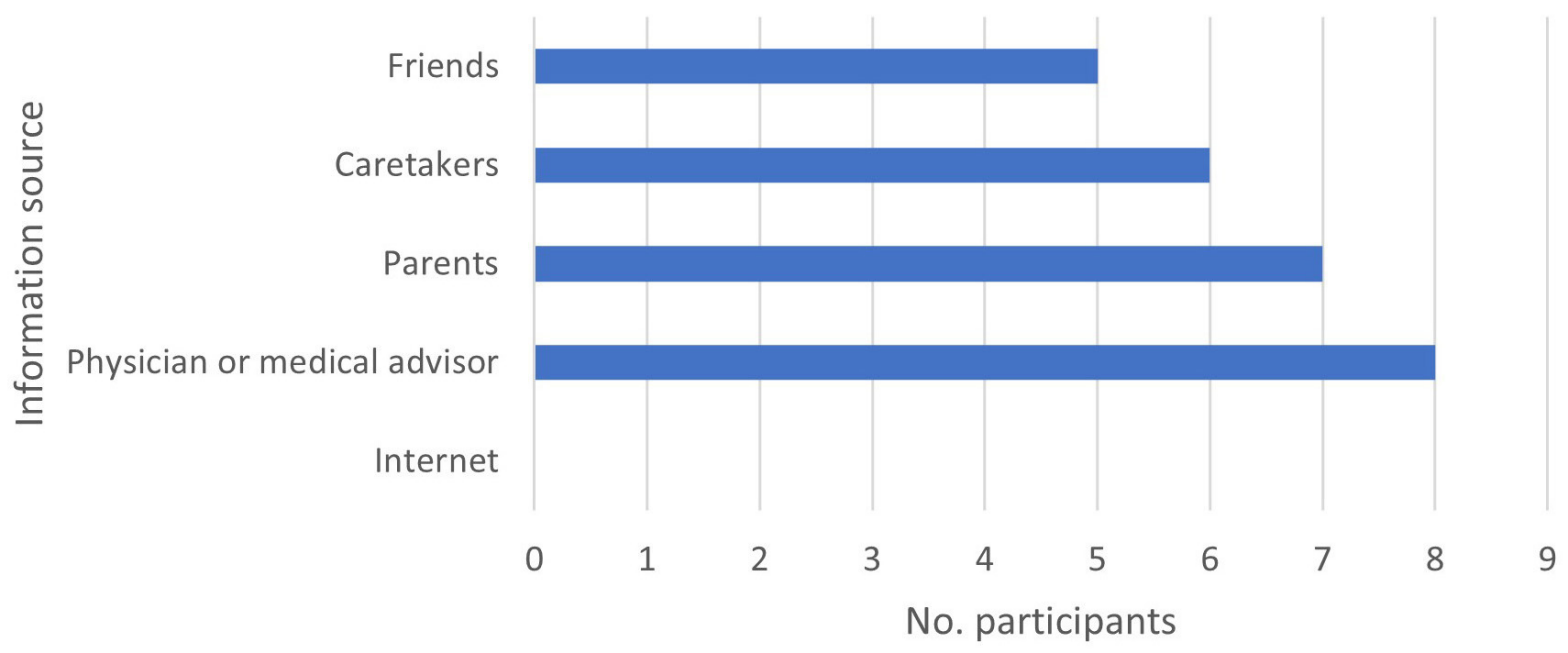
Source of health information of participants with mild to moderate intellectual disabilities.

Participants were equally distributed by gender (5 male, 5 female), ranging from 16 to 53 years of age. When participants were asked about their familiarity with and the usage of computers and the internet, most participants knew how. Although half of the participants used the internet, two needed assistance to navigate confidently. As shown in Figure 1, most participants acquire health information from their physician or a medical professional.

**Figure 2:**
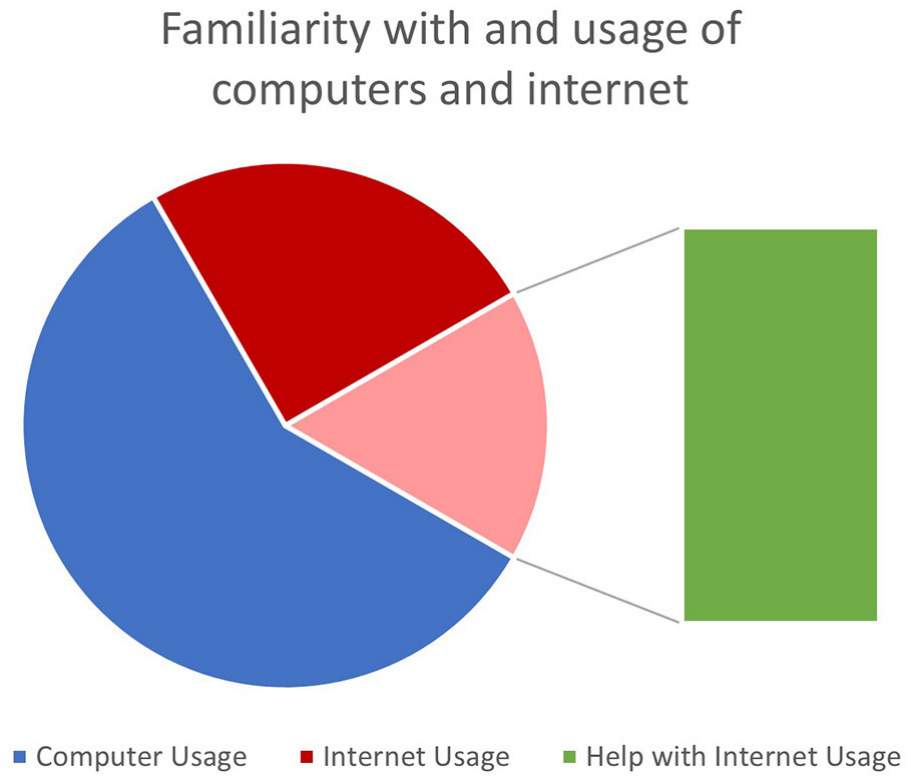
Number of correct answers per question.

As illustrated in Figure 2, all participants answered the nutrition and first aid questions correctly. Furthermore, most participants were familiar with the medical specialization. However, none of the participants knew what ELGA was supposed to be, and participants had great difficulties understanding the purpose of the question.

The test run results showed that some questions were too complicated or too complex. Especially questions where the correct answer was “No” and questions where it was necessary to associate different words with each other posed the greatest barriers. Furthermore, it was noticeable that participants were rather insecure initially, though all participants but one got more comfortable with the researcher by no later than the last two questions about first aid. Finally, the participants recounted their own experiences, and almost every participant had participated in a first aid course and shared their experiences during their training.

**Figure 3:**
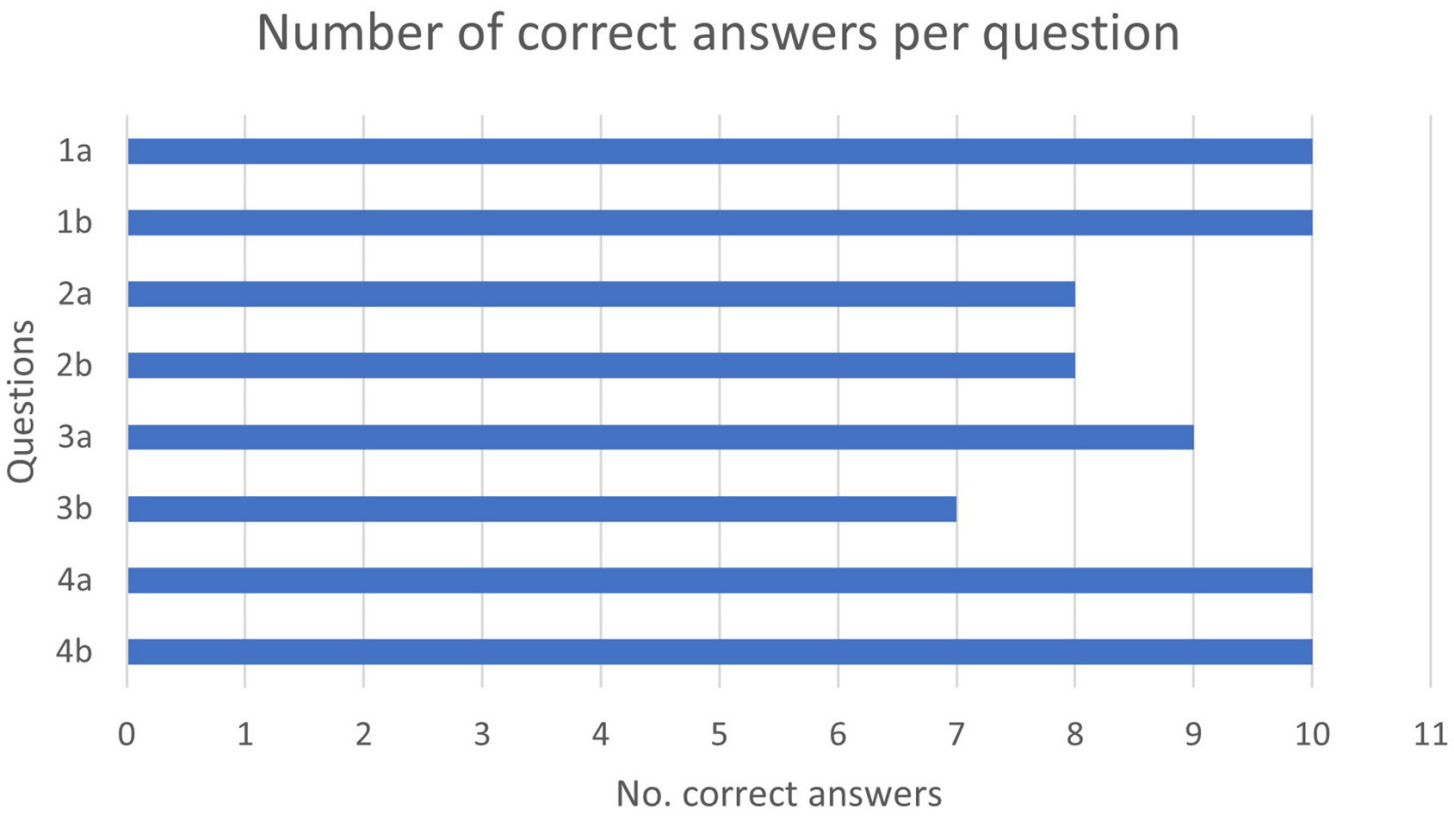
Number of correct answers per participant.

Figure 3 shows the number of correctly answered questions per participant. Most participants answered every question correctly. The most difficult questions are 1b, 2b, and 3b, whereas the easiest questions are 1a, 4a, and 4b, rated by a point scale to record their achievements. The assigned points reflect the results regarding the questions about nutrition and first aid and question 3b about the Austrian electronic health record. The dichotomous answers allowed only to gain all points per question or none.

## Discussion

Our results show that completing the questionnaire without support was difficult for the participants since they were often insecure and had queries. Although this was not required, most subjects read the articles and questions aloud, used long pauses in their reading flow, or stared blankly at the questionnaire to communicate their need for further help. If such a situation occurred, the researcher instantly knew where a participant needed assistance without actively asking for it, and immediate support could be provided. Almost all the participants could answer the questions verbally correctly but had to be reminded that they needed to select the correct answer on the questionnaire, which posed a challenge to the participants. In addition, most participants showed that they are easily distracted by their need to share their experiences triggered by the questionnaire.

The project LUCHS aims to develop an appropriate questionnaire for people with ID to measure the improvement of health literacy and empowerment of people with intellectual disabilities. Following guidelines for easy-to-read language and web accessibility, the team created a website containing health information tailored to the interests of the participants. The website helped the participants navigate the questionnaire, although additional help from supervising investigators was necessary. Due to the heterogeneity of intellectual disabilities, replicating this community case study will most likely result in different findings (32). A potential inconsistency in a replicated conduction of the study might be strongly affected by high inter-day variability of the individual condition of the participants, influenced by factors such as medication. Despite highly sensitive variables related to intellectual disabilities, this pilot study shows that a questionnaire is feasible to measure the knowledge transfer of people with intellectual disabilities; however, multiple versions adapted to the heterogeneous participants might be necessary to reach a larger group.

Pre-tests with participants with another questionnaire might be helpful in this process to gain experience filling out questionnaires and reduce possible anxiety and tension. All questionnaires introduced should adhere to the guidelines of easy-to-read language and be presented in a similar format. If researchers are present during the intervention, the authors recommend a relaxed atmosphere to become acquainted with the participants to reduce possible anxiety and satisfy curiosity. In order to include illiterate people with ID, semi-structured interviews with expressive language (33) can capture the perspective and lived realities (34), allow in-depth contextual and relevant data to be attained from the target population (35) and include a broader group in health education research. An alternative could be using artificial intelligence (AI) to read questions aloud and record the participants' answers. However, this seems to be a time and cost-intensive possibility. A simpler start would be tests and training with yes-no questions. This would enhance participants' technical skills and improve the AI's understanding of multiple dialects and pronunciations.

Almost all participants could answer the question verbally correctly but had to be reminded that they needed to select the correct answer on the questionnaire, which posed a challenge to the participants. In addition, most participants showed that they are easily distracted by their need to share their experiences triggered by the questionnaire.

As mentioned, the intellectual disability of the target group can be categorized by F70 and F71 in the ICD-10. F70 equals mild mental retardation; the IQ ranges between 50 and 69. F71 equals moderate mental retardation with the IQ ranging from 35 to 49 (11, 21, 36) (pp. 236–237). Several studies have reported that low attentional capacity is a characteristic of mental retardation (12, 36). However, no information about the approximate attention span of people with mild to moderate mental retardation was found, though it can be assumed that moderate to strong correlation between moderate intellectual disability and lower attention span (37).

One unexpected problem was that one participant's physical and mental condition on the test day hindered the questionnaire completion. The research team was unaware that on the day of the investigation, the physical and mental conditions of the participants might vary to an impacting extent. A caretaker informed the researcher of this fact at the end of the investigation. As a result, the duration of most investigations was longer than expected since they each required over 30 min, especially on the first day of the investigation. The investigations on the second day took between 20 and 30 min.

Another unexpected problem was that the participants needed more assistance than anticipated. The research team needed to read the questions aloud or repeat parts or the whole question in proper German. Since they are easily distractible, it was important to remind the individuals to answer the questions instead of telling stories. As soon as questions triggered memories of experiences, the participants started recounting them to the research team, so the investigation lasted longer. For example, two participants spent time discussing their perception of cheese, and it took a long time for them to answer the question. A major problem with more than half of the participants was that they verbally answered the question correctly but ticked the wrong checkbox. Although answering only required ticking a checkbox, they seemed to have difficulties committing their verbal answers to paper. The team alerted them of their mistake and let them correct it. One challenge occurred with the layout of the questionnaire. The font size was appropriate for the participants. However, the research team noted that two subjects had very weak eyesight and that they would have profited from a second questionnaire with larger font size (38). A challenge for the team was the lack of communication from one participant since it was hardly possible to gauge whether the participant understood everything or needed assistance. The individual did not ask questions, read aloud, or make eye contact. Therefore, the research team recommends the presence of confidential caretakers for further studies or replications.

## Conclusion

Based on the outcomes of this study, a structured questionnaire may not be the most efficient tool to measure knowledge transfer in health-related topics for people with intellectual disabilities. The authors suggest that semi-structured interviews with an appropriately trained and briefed interviewer and an accompanying questionnaire may be a more successful approach to such assessments due to greater flexibility and higher individualization. Based on the evaluation of the process, we assume that assessing knowledge in people with intellectual disabilities might be more comfortable within a conversation. Furthermore, the results of this study showed the impact and usefulness of images on the comprehension of the subject matter since none of the participants needed to read the article to know what was displayed. Formed upon these experiences, the next step should aim to improve the website and the questionnaire, particularly in phrasing and compound words.

The authors recommend further investigations regarding people's attention spans with intellectual disabilities as categorized by ICD-11 since hardly any scientific literature was found. In order to obtain a scientifically sound and reliable tool for measuring knowledge transfer, further research as pilot tests are necessary. Moreover, the authors recommend cooperation with special education teachers since they might give valuable insight into examining knowledge of this particular target group. In addition, confidentiality could support the investigation, reduce insecurity, and support the overall process. Finally, the design of an improved questionnaire would lead to more scientific relevant data. In conclusion, this case study points out potential barriers people with intellectual disabilities face in knowledge acquisition and application and urges for more inclusiveness in health-related and educational research.

Furthermore, our results highlight the importance of developing inclusive strategies to improve health-related knowledge transfer to enhance health literacy and related outcomes in people with mental impairments.

## Data availability statement

The datasets presented in this study can be found in online repositories. The names of the repository/repositories and accession number(s) can be found in the article/supplementary material.

## Ethics statement

The studies involving human participants were reviewed and approved by Ethics Committee of Lower Austria. The patients/participants provided their written informed consent to participate in this study.

## Author contributions

All authors have contributed equally and approved it for publication.

## Conflict of interest

The authors declare that the research was conducted in the absence of any commercial or financial relationships that could be construed as a potential conflict of interest.

## Publisher's note

All claims expressed in this article are solely those of the authors and do not necessarily represent those of their affiliated organizations, or those of the publisher, the editors and the reviewers. Any product that may be evaluated in this article, or claim that may be made by its manufacturer, is not guaranteed or endorsed by the publisher.

## Footnotes

^1^“Leicht Lesen” means “easy-to-read” and it is a quality seal for texts in the German language (capito, no date).
